# Supplementary material for: 1–2 Drinks Per Day Affect Lipoprotein Composition after 3 Weeks—Results from a Cross-Over Pilot Intervention Trial in Healthy Adults Using Nuclear Magnetic Resonance-Measured Lipoproteins and Apolipoproteins
Source: Nutrients. 2022 Nov 27;14(23):5043. doi: 10.3390/nu14235043 (PMC9735490; doi:10.3390/nu14235043)
Supplement: Supplementary file 1 [file nutrients-14-05043-s001.zip › nutrients-1901824-supplementary.pdf]

Supplementary Material

1 Supplementary Methods

1.1 Chemicals

Chemicals and reagents used in this study were purchased from Sigma-Aldrich (Søborg, Denmark). They included deuterium oxide (D_2O, 99.9 atom % D), sodium phosphate monobasic monohydrate (NaH_2PO_3, H_2O), sodium phosphate dibasic heptahydrate (Na_2HPO_3, 7 H_2O), monobasic potassium phosphate (KH_2PO_4, ≥99.0%), dibasic potassium phosphate (K_2HPO_4, ≥98.0%), sodium salt of 3-(trimethylsilyl) propionic-2,2,3,3-d4 acid (TSP, 98 atom % D, ≥98.0%), and sodium azide (NaN_3, ≥99.5%). The water used throughout the study was purified using a Millipore lab water system (Merck KGaA, Darmstadt, Germany) equipped with a 0.22 μm filter membrane.

1.2 Blood plasma sample preparation for NMR measurements

Sample preparation was performed according to the standard operating procedures described by Dona et al. and Monsonis at al.(34,35). Briefly, for each sample, 350 μl phosphate buffer solution(36) containing TSP (5 mM) and D_2O (20 %) were transferred to a 2 ml Eppendorf tube and gently mixed with equal amounts of plasma. Aliquots of 600 μl were then transferred into 5 mm O.D. (103.5 mm) NMR tubes. Samples were loaded into SampleJet racks (96 well plate format for batch operation) (Bruker BioSpin, Ettlingen, Germany) and measured within 72h after preparation.

2 Supplementary Results

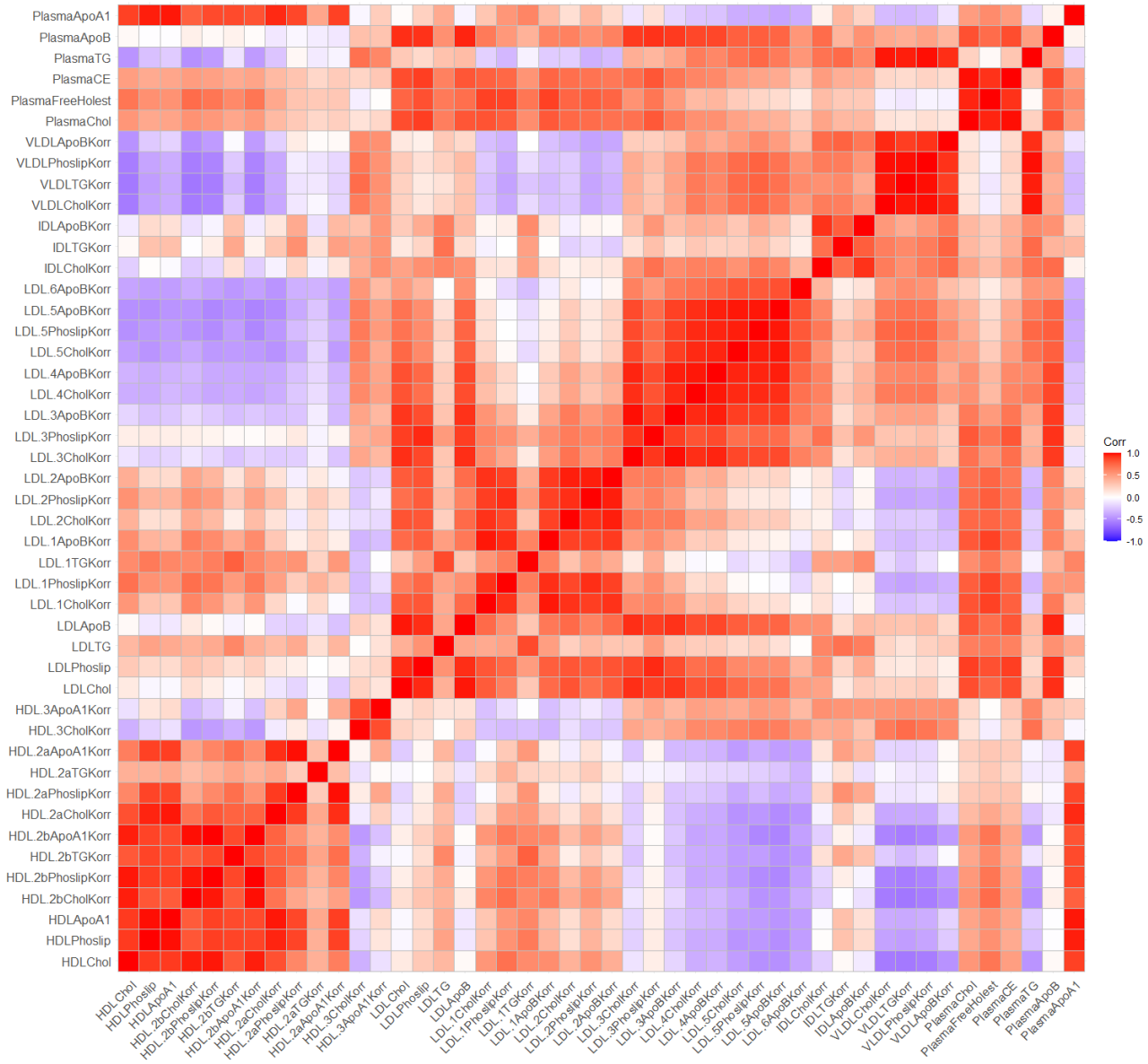

Figure S1. Correlations among lipoproteins and lipoprotein subfractions.
Lipoporoteins defined by their apoB content is converted to particle number in the main analyses (Table S2).
Apo: apolipoprotein, CE: cholesterol ester, HDL: high-density lipoprotein, IDL: intermediate-density lipoprotein LDL: low-density lipoprotein PL: phospholipid, TG: triglyceride, VLDL: very-low-density lipoprotein.

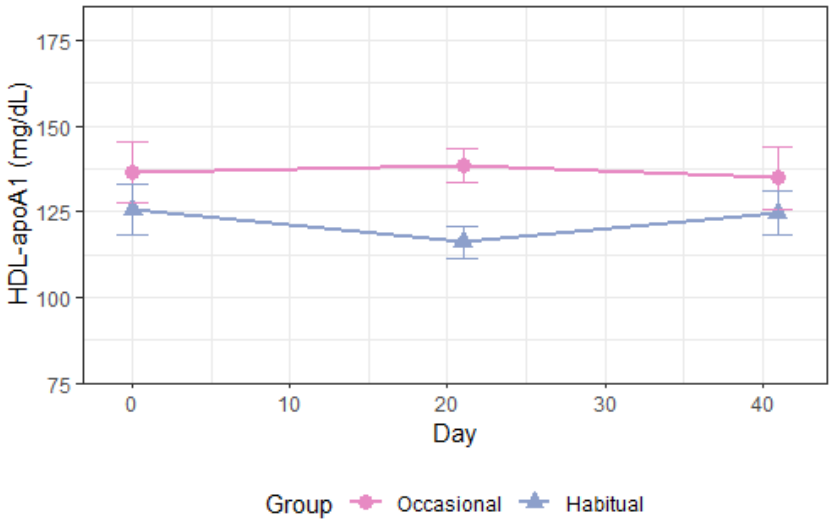

Figure S2. Circulating HDL-apoA1 during 3 weeks in period 1 and 3 weeks in period 2.
Occasional drinkers (n = 10): habitual alcohol intake < 2 drinks (~24 g) per week.
Habitual drinkers (n = 16): habitual alcohol intake ≥ 2 drinks (~24 g) per week, max 1 drink per day in women and 2 drinks per day in men.
Apo: apolipoprotein, HDL: high-density lipoprotein.

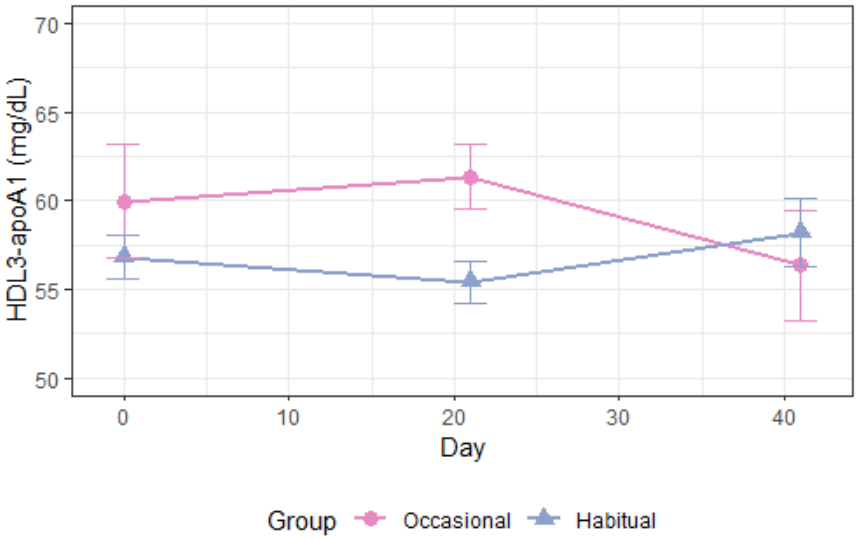

Figure S3. Circulating HDL_3-apoA1 during 3 weeks in period 1 and 3 weeks in period 2.
Occasional drinkers (n = 10): habitual alcohol intake < 2 drinks (~24 g) per week.
Habitual drinkers (n = 16): habitual alcohol intake ≥ 2 drinks (~24 g) per week, max 1 drink per day in women and 2 drinks per day in men.
Apo: apolipoprotein, HDL: high-density lipoprotein.

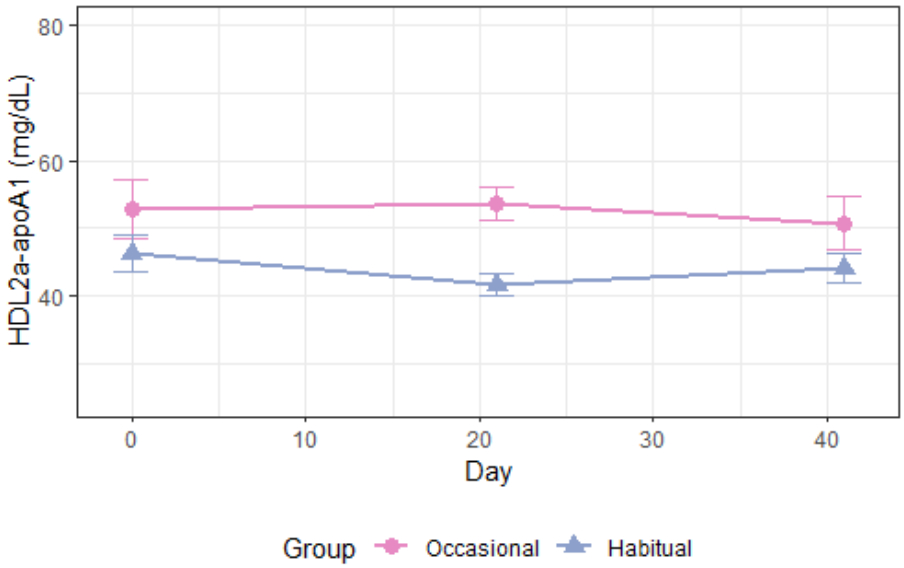

Figure S4. Circulating HDL_{2a}-apoA1 during 3 weeks in period 1 and 3 weeks in period 2.
Occasional drinkers (n = 10): habitual alcohol intake < 2 drinks (~24 g) per week.
Habitual drinkers (n = 16): habitual alcohol intake ≥ 2 drinks (~24 g) per week, max 1 drink per day in women and 2 drinks per day in men.
Apo: apolipoprotein, HDL: high-density lipoprotein.

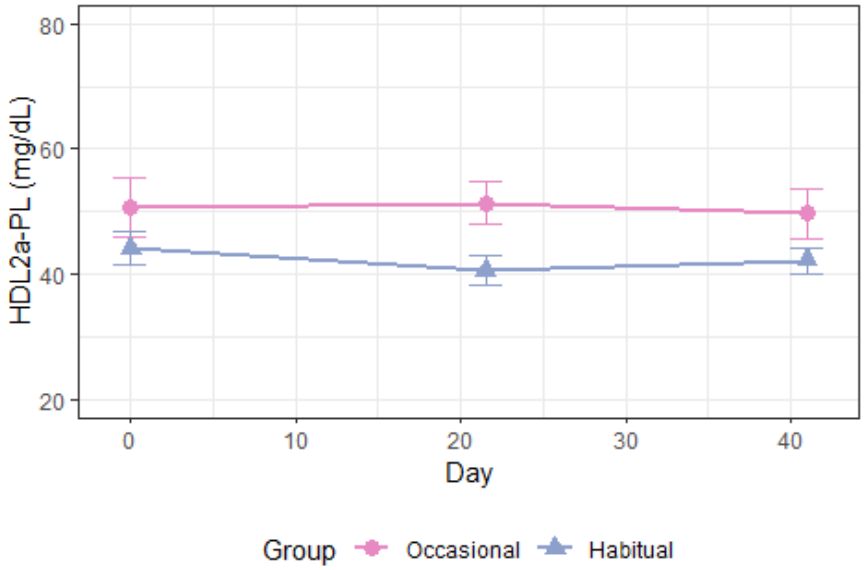

Figure S5. Circulating HDL_{2a}-PL during 3 weeks in period 1 and 3 weeks in period 2.
Occasional drinkers (n = 10): habitual alcohol intake < 2 drinks (~24 g) per week.
Habitual drinkers (n = 16): habitual alcohol intake ≥ 2 drinks (~24 g) per week, max 1 drink per day in women and 2 drinks per day in men.
Apo: apolipoprotein, HDL: high-density lipoprotein, -PL: phospholipid concentration

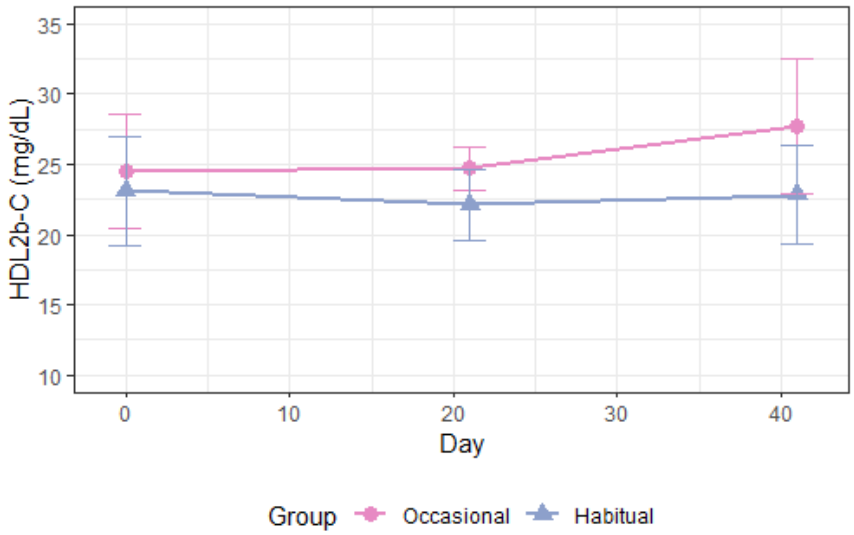

Figure S6. Circulating HDL_{2b}-C during 3 weeks in period 1 and 3 weeks in period 2.
Occasional drinkers (n = 10): habitual alcohol intake < 2 drinks (~24 g) per week.
Habitual drinkers (n = 16): habitual alcohol intake ≥ 2 drinks (~24 g) per week, max 1 drink per day in women and 2 drinks per day in men.
-C: cholesterol concentration, HDL: high-density lipoprotein.

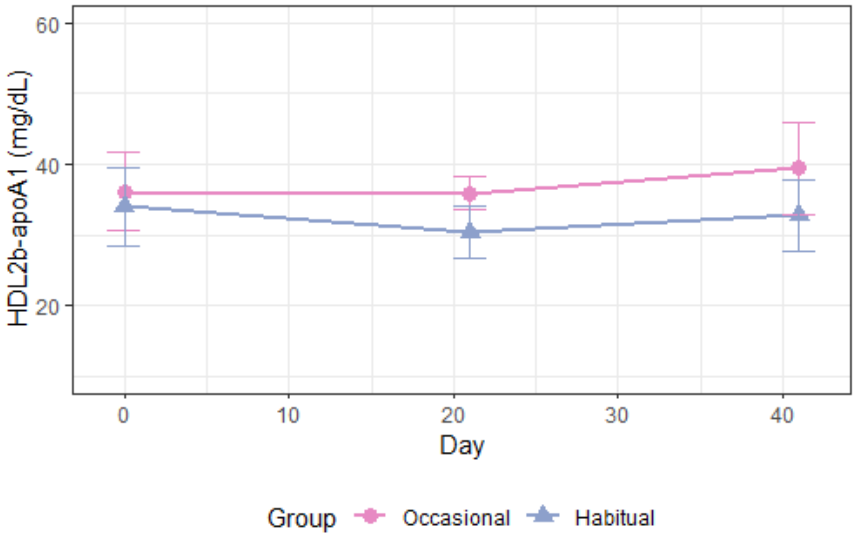

Figure S7. Circulating HDL_{2b}-apoA1 during 3 weeks in period 1 and 3 weeks in period 2.
Occasional drinkers (n = 10): habitual alcohol intake < 2 drinks (~24 g) per week.
Habitual drinkers (n = 16): habitual alcohol intake ≥ 2 drinks (~24 g) per week, max 1 drink per day in women and 2 drinks per day in men.
Apo: apolipoprotein, HDL: high-density lipoprotein.

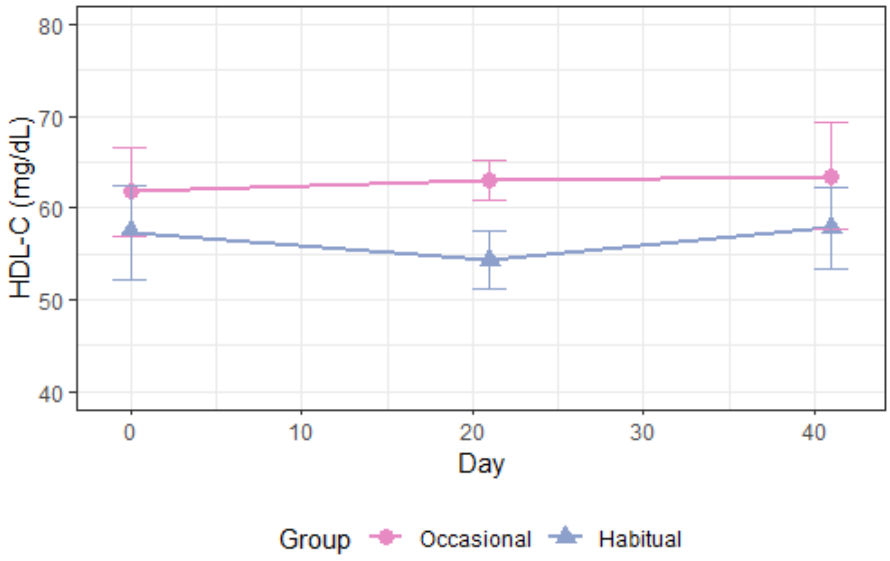

Figure S8. Circulating HDL-C during 3 weeks in period 1 and 3 weeks in period 2.
Occasional drinkers (n = 10): habitual alcohol intake < 2 drinks (~24 g) per week.
Habitual drinkers (n = 16): habitual alcohol intake ≥ 2 drinks (~24 g) per week, max 1 drink per day in women and 2 drinks per day in men.
-C: cholesterol concentration, HDL: high-density lipoprotein.

Table S1. Effects of 1-2 drinks (~12-24 g) per day on NMR measured whole plasma lipids before and after alcohol intake or abstention and in drinking periods compared with abstention.

	Period 1			Period 2			Period 1		Period 2		Period 1 + 2	
	**Day 0**	**Day 21**	**P**	**Day 22**	**Day 42**	**P**	**ΔMean1[£]**	**P**	**ΔMean2[¤]**	**P**	**ΔMean drinking vs. abstention***	**P**
	Mean (SD)	Mean (SD)		Mean (SD)	Mean (SD)		Mean (95% CI)		Mean (95% CI)		Mean (95% CI)	
**TG,** mg/dL												
Occ. drinkers	100.1 (±33.4)[$]	90.6 (±30.4)[$]	0.28	90.6 (±30.4)[#]	91.7 (±44.8)[#]	0.92						
Hab. drinkers	104.9 (±45.8)[#]	102.9 (±55.6)[#]	0.86	102.9 (±55.6)[$]	120.8 (±51.4)[$]	0.07						
Occ. vs. Hab.							7.7 (-16.4-31.8)	0.53	-16.8 (-40.7-7.2)	0.17		
All											-4.5 (-21.5-12.4)	0.60
**TC,** mg/dL												
Occ. drinkers	183.3 (±28.1)[$]	184.8 (±26.6)[$]	0.80	184.8 (±26.6)[#]	190.1 (±35.3)[#]	0.63						
Hab. drinkers	179.1 (±33.2)[#]	191.6 (±52.3)[#]	0.58	191.6 (±52.3)[$]	199.0 (±49.8)[$]	0.20						
Occ. vs. Hab.							3.4 (-15.6-22.4)	0.73	-2.1 (-21.0-16.7)	0.83		
All											0.6 (-12.8-14.0)	0.93
**FC,** mg/dL												
Occ. drinkers	56.7 (±8.9)[$]	56.2 (±6.7)[$]	0.76	56.2 (±6.7)[#]	58.7 (±9.7)[#]	0.36						
Hab. drinkers	54.9 (±9.7)[#]	58.6 (±13.3)[#]	0.39	58.6 (±13.3)[$]	61.0 (±12.5)[$]	0.09						
Occ. vs. Hab.							2.2 (-2.4-6.9)	0.35	0.2 (-4.5-4.8)	0.95		
All											1.2 (-2.1-4.5)	0.47
**CE,** mg/dL												
Occ. drinkers	215.4 (±32.4)[$]	218.6 (±32.0)[$]	0.67	218.6 (±32.0)[#]	223.4 (±43.5)[#]	0.74						
Hab. drinkers	211.2 (±38.8)[#]	225.4 (±64.2)[#]	0.67	225.4 (±64.2)[$]	233.3 (±60.9)[$]	0.31						
Occ. vs. Hab.							1.7 (-23.2-26.6)	0.89	-3.1 (-27.8-21.6)	0.81		
All											-0.7 (-18.2-16.8)	0.94
**PL,** mg/dL												
Occ. drinkers	242.2 (±41.0)[$]	241.9 (±29.5)[$]	0.96	241.9 (±29.5)[#]	245.0 (±47.1)[#]	0.82						
Hab. drinkers	227.1 (±39.6)[#]	229.7 (±44.2)[#]	0.71	229.7 (±44.2)[$]	241.8 (±38.8)[$]	0.08						
Occ. vs. Hab.							-2.2 (-24.1-19.6)	0.84	-8.9 (-30.6-12.7)	0.42		
All											-5.6 (-21.0-9.8)	0.48

Occasional drinkers (n = 10): habitual alcohol intake < 2 drinks (~24 g) per week.
Habitual drinkers (n = 16): habitual alcohol intake ≥ 2 drinks (~24 g) per week, max 1 drink per day in women and 2 drinks per day in men.
[$]Drinking (three weeks).
[#]Abstention (three weeks).
[£]ΔMean1: mean change in occasional drinkers compared with mean change in habitual drinkers in period 1.
[¤]ΔMean2: mean change in occasional drinkers compared with mean change in habitual drinkers in period 2.
*Mean changes in lipids and lipoproteins in drinking participants compared with abstaining participants in both periods, independent of group relationship.
[£,¤,]*Values for drinking periods were subtracted from abstaining, and negative numbers therefore indicate increased levels while drinking compared with abstaining.
CE: cholesteryl ester, FC: free cholesterol, Hab.: habitual, Occ.: occasional, PL: phospholipids, TC: total cholesterol, TG: triglycerides.

Table S2. Effects of 1-2 drinks (~12-24 g) per day on NMR measured HDLs, apoA1, and HDL subfractions before and after alcohol intake or abstention and in drinking periods compared with abstention – absolute levels before and after interventions in periods 1-2.

	Period 1			Period 2		
	**Day 0**	**Day 21**	**P**	**Day 22**	**Day 42**	**P**
	Mean (SD)	Mean (SD)		Mean (SD)	Mean (SD)	
**HDL-C,** mg/dL						
Occ. drinkers	61.8 (±15.2)$	63.0 (±9.8)$	0.76	63.0 (±9.8)#	63.5 (±18.5)#	0.94
Hab. drinkers	57.3 (±19.7)#	54.3 (±18.2)#	0.24	54.3 (±18.2)$	57.9 (±17.8)$	0.08
**HDL_{2b}-C,** mg/dL						
Occ. drinkers	24.5 (±13.0)$	24.7 (±7.3)$	0.95	24.7 (±7.3)#	27.7 (±15.3)#	0.41
Hab. drinkers	23.1 (±15.2)#	22.1 (±14.5)#	0.57	22.1 (±14.5)$	22.8 (±13.9)$	0.56
**HDL_{2a}-C,** mg/dL						
Occ. drinkers	25.0 (±5.0)$	25.7 (±4.2)$	0.40	25.7 (±4.2)#	24.9 (±4.9)#	0.60
Hab. drinkers	22.7 (±5.2)#	21.0 (±4.6)#	**0.01**	21.0 (±4.6)$	22.1 (±4.8)$	0.07
**HDL_3-C,** mg/dL						
Occ. drinkers	18.0 (±2.7)$	19.1 (±1.9)$	0.22	19.1 (±1.9)#	17.5 (±2.2)#	**0.03**
Hab. drinkers	18.1 (±1.6)#	17.8 (±2.0)#	0.38	17.8 (±2.0)$	17.8 (±2.8)$	0.98
**HDL_{2b}-TG,** mg/dL						
Occ. drinkers	4.0 (±1.4)$	3.6 (±1.0)$	0.30	3.6 (±1.0)#	4.0 (±1.3)#	0.20
Hab. drinkers	3.7 (±1.5)#	3.4 (±1.5)#	0.66	3.4 (±1.5)$	3.9 (±1.3)$	**0.01**
**HDL_{2a}-TG,** mg/dL						
Occ. drinkers	4.1 (±0.6)$	3.9 (±0.6)$	0.52	3.9 (±0.8)#	4.0 (±0.9)#	0.70
Hab. drinkers	4.1 (±0.8)#	3.9 (±1.1)#	0.62	3.9 (±1.1)$	4.7 (±0.7)$	**0.0001**
**HDL-PL,** mg/dL						
Occ. drinkers	115.8 (±29.8)$	116.1 (±21.6)$	0.96	116.1 (±21.6)#	116.4 (±28.8)#	0.97
Hab. drinkers	101.5 (±32.6)#	94.3 (±28.4)#	0.17	94.3 (±28.4)$	100.1 (±27.9)$	0.10
**HDL_{2b}-PL,** mg/dL						
Occ. drinkers	41.6 (±20.9)$	41.4 (±12.2)$	0.96	41.4 (±12.2)#	45.4 (±23.9)#	0.52
Hab. drinkers	37.3 (±25.7)#	33.8 (±24.5)#	0.38	33.8 (±24.5)$	36.8 (±23.5)$	0.18
**HDL_{2a}-PL,** mg/dL						
Occ. drinkers	50.7 (±14.7)$	51.4 (±11.0)$	0.72	51.4 (±11.0)#	49.6 (±12.5)#	0.59
Hab. drinkers	44.1 (±10.5)#	40.6 (±9.9)#	**0.03**	40.6 (±9.9)$	42.1 (±8.5)$	0.33
**ApoA1,** mg/dL						
Occ. drinkers	157.9 (±30.7)$	159.8 (±24.8)$	0.71	159.8 (±24.8)#	152.8 (±33.4)#	0.49
Hab. drinkers	148.2 (±28.5)#	137.3 (±26.8)#	**0.03**	137.3 (±26.8)$	145.8 (±24.8)$	**0.02**
**HDL-apoA1,** mg/dL						
Occ. drinkers	136.5 (±27.6)$	138.5 (±21.8)$	0.68	138.5 (±21.8)#	134.9 (±29.5)#	0.69
Hab. drinkers	125.6 (±28.9)#	116.2 (±26.8)#	**0.03**	116.2 (±26.8)$	124.7 (±25.1)$	**0.01**
**HDL_{2b}-apoA1,** mg/dL						
Occ. drinkers	36.1 (±17.6)$	35.8 (±10.7)$	0.95	35.8 (±10.7)#	39.4 (±20.9)#	0.49
Hab. drinkers	34.0 (±21.5)#	30.4 (±21.4)#	0.32	30.4 (±21.4)$	32.7 (±20.1)$	0.20
**HDL_{2a}-apoA1,** mg/dL						
Occ. drinkers	52.8 (±13.8)$	53.6 (±11.4)$	0.59	53.6 (±11.4)#	50.8 (±12.2)#	0.36
Hab. drinkers	46.3 (±10.5)#	41.7 (±9.8)#	**0.004**	41.7 (±9.8)$	44.1 (±8.8)$	0.06
**HDL_3-apoA1,** mg/dL						
Occ. drinkers	60.0 (±10.2)$	61.3 (±8.5)$	0.49	61.3 (±8.5)#	56.3 (±9.8)#	0.051
Hab. drinkers	56.8 (±4.8)#	55.4 (±6.7)#	0.24	55.4 (±6.7)$	58.2 (±7.8)$	0.11
**ApoB/apoA1,** mg/dL						
Occ. drinkers	0.56 (±0.13)$	0.54 (±0.14)$	0.66	0.54 (±0.14)#	0.58 (±0.13)#	0.33
Hab. drinkers	0.58 (±0.17)#	0.69 (±0.22)#	**0.04**	0.69 (±0.22)$	0.66 (±0.20)$	0.058

Occasional drinkers (n = 10): habitual alcohol intake < 2 drinks (~24 g) per week.

Habitual drinkers (n = 16): habitual alcohol intake ≥ 2 drinks (~24 g) per week, max 1 drink per day in women and 2 drinks per day in men.

$Drinking (three weeks).

#Abstention (three weeks).

Apo: apolipoprotein, -C: cholesterol concentration, Hab: habitual, HDL: high-density lipoprotein, Occ: occasional, -PL: phospholipid concentration, -TG: triglyceride concentration.

Table S3. Effects of 1-2 drinks (~12-24 g) per day on NMR measured LDLs, apoB, and LDL subfractions before and after alcohol intake or abstention and in drinking periods compared with abstention.

	Period 1			Period 2			Period 1		Period 2		Period 1 + 2	
	**Day 0**	**Day 21**	**P**	**Day 22**	**Day 42**	**P**	**ΔMean1**[£]	**P**	**ΔMean2**[¤]	**P**	**ΔMean drinking vs. abstention***	**P**
	Mean (SD)	Mean (SD)		Mean (SD)	Mean (SD)		Mean (95% CI)		Mean (95% CI)		Mean (95% CI)	
**LDL-C,** mg/dL												
Occ. drinkers	90.7 (±17.6)[$]	92.6 (±20.2)[$]	0.71	92.6 (±20.2)[#]	95.9 (±17.1)[#]	0.63						
Hab. drinkers	89.9 (±22.0)[#]	103.6 (±42.5)[#]	0.32	103.6 (±42.5)[$]	104.7 (±38.9)[$]	0.78						
Occ. vs. Hab.							5.5 (-8.8-19.9)	0.45	2.1 (-12.1-16.4)	0.77		
All											3.8 (-6.3-13.9)	0.46
**LDL$_1$-C,** mg/dL												
Occ. drinkers	23.0 (±7.6)[$]	23.7 (±8.4)[$]	0.76	23.7 (±8.4)[#]	26.0 (±6.1)[#]	0.34						
Hab. drinkers	23.1 (±8.8)[#]	28.0 (±16.2)[#]	0.30	28.0 (±16.2)[$]	28.2 (±14.3)[$]	0.91						
Occ. vs. Hab.							1.8 (-3.0-6.6)	0.45	2.2 (-2.5-7.0)	0.36		
All											2.0 (-1.3-5.4)	0.24
**LDL$_2$-C,** mg/dL												
Occ. drinkers	14.2 (±3.1)[$]	15.0 (±3.9)[$]	0.52	15.0 (±3.9)[#]	15.3 (±2.5)[#]	0.83						
Hab. drinkers	14.0 (±4.0)[#]	15.8 (±8.7)[#]	0.75	15.8 (±8.7)[$]	15.8 (±8.0)[$]	0.92						
Occ. vs. Hab.							-0.2 (-3.2-2.7)	0.87	0.3 (-2.6-3.3)	0.82		
All											0.05 (-2.0-2.1)	0.96
**LDL$_3$-C,** mg/dL												
Occ. drinkers	13.5 (±2.8)[$]	13.8 (±3.1)[$]	0.76	13.8 (±3.1)[#]	14.0 (±3.0)[#]	0.85						
Hab. drinkers	12.9 (±3.8)[#]	15.1 (±6.9)[#]	0.37	15.1 (±6.9)[$]	15.6 (±6.4)[$]	0.49						
Occ. vs. Hab.							0.9 (-1.6-3.4)	0.47	-0.3 (-2.8-2.2)	0.82		
All											0.3 (-1.5-2.1)	0.72
**LDL$_4$-C,** mg/dL												
Occ. drinkers	13.1 (±2.3)[$]	13.4 (±2.7)[$]	0.70	13.4 (±2.7)[#]	13.2 (±3.1)[#]	0.88						
Hab. drinkers		14.5 (±5.7)[#]	0.52	14.5 (±5.7)[$]	14.8 (±5.5)[$]	0.62						
Occ. vs. Hab.	13.0 (±3.5)[#]						0.5 (-1.8-2.7)	0.69	-0.5 (-2.7-1.8)	0.69		
All											0.003 (-1.6-1.6)	0.99
**LDL$_5$-C,** mg/dL												
Occ. drinkers	10.3 (±2.7)[$]	10.2 (±2.5)[$]	0.84	10.2 (±2.5)[#]	10.3 (±2.9)[#]	0.81						
Hab. drinkers	10.3 (±3.5)[#]	11.6 (±4.6)[#]	0.31	11.6 (±4.6)[$]	12.3 (±4.7)[$]	0.28						
Occ. vs. Hab.							1.0 (-0.8-2.8)	0.27	-0.5 (-2.3-1.2)	0.56		
All											0.2 (-1.0-1.5)	0.70
**LDL-TG,** mg/dL												
Occ. drinkers	16.1 (±3.3)[$]	15.9 (±2.1)[$]	0.83	15.9 (±2.1)[#]	16.8 (±3.6)[#]	0.38						
Hab. drinkers	14.4 (±3.1)[#]	16.4 (±4.1)[#]	**0.02**	16.4 (±4.1)[$]	16.8 (±3.2)[$]	0.62						
Occ. vs. Hab.							2.1 (0.05-4.1)	**0.04**	0.6 (-1.4-2.5)	0.58		
All											1.3 (-0.1-2.7)	0.070
**LDL$_1$-TG,** mg/dL												
Occ. drinkers	5.4 (±1.9)[$]	5.2 (±0.9)[$]	0.76	5.2 (±0.9)[#]	5.5 (±1.7)[#]	0.50						
Hab. drinkers	4.4 (±1.8)[#]	5.3 (±2.2)[#]	**0.047**	5.3 (±2.2)[$]	5.3 (±1.8)[$]	1.00						
Occ. vs. Hab.							1.0 (-0.05-2.0)	0.06	0.3 (-0.7-1.3)	0.53		
All											0.6 (-0.1-1.4)	0.076
**LDL-PL,** mg/dL												
Occ. drinkers	64.5 (±11.9)[$]	66.0 (±12.3)[$]	0.67	66.0 (±12.3)[#]	68.4 (±12.4)[#]	0.61						
Hab. drinkers	60.2 (±14.5)[#]	70.5 (±30.2)[#]	0.25	70.5 (±30.2)[$]	69.8 (±26.2)[$]	0.82						
Occ. vs. Hab.							4.5 (-5.8-14.9)	0.39	3.2 (-7.1-13.4)	0.55		
All											3.8 (-3.4-11.1)	0.30
**LDL$_1$-PL,** mg/dL												
Occ. drinkers	15.7 (±4.4)[$]	16.3 (±4.3)[$]	0.69	16.3 (±4.3)[#]	17.4 (±4.3)[#]	0.49						
Hab. drinkers	14.2 (±6.6)[#]	18.3 (±10.1)[#]	0.37	18.3 (±10.1)[$]	18.0 (±9.2)[$]	0.62						
Occ. vs. Hab.							1.2 (-2.5-4.9)	0.52	0.65 (-3.0-4.3)	0.73		
All											0.9 (-1.7-3.5)	0.48
**LDL$_2$-PL,** mg/dL												
Occ. drinkers	9.3 (±2.0)[$]	9.5 (±1.9)[$]	0.70	9.5 (±1.9)[#]	9.5 (±2.3)[#]	0.96						
Hab. drinkers	8.4 (±2.7)[#]	10.2 (±4.8)[#]	0.44	10.2 (±4.8)[$]	9.7 (±4.9)[$]	0.94						
Occ. vs. Hab.							0.4 (-1.4-2.1)	0.67	0.06 (-1.7-1.8)	0.95		
All											0.2 (-1.0-1.5)	0.73
**LDL$_3$-PL,** mg/dL												
Occ. drinkers	9.6 (±1.9)[$]	9.7 (±1.8)[$]	0.84	9.7 (±1.8)[#]	10.0 (±2.1)[#]	0.73						
Hab. drinkers	8.7 (±2.2)[#]	10.3 (±4.4)[#]	0.23	10.3 (±4.4)[$]	10.3 (±3.9)[$]	0.99						
Occ. vs. Hab.							0.9 (-0.8-2.5)	0.30	0.3 (-1.4-1.9)	0.76		
All											0.6 (-0.6-1.7)	0.34
**LDL$_5$-PL,** mg/dL												
Occ. drinkers	7.7 (±1.7)[$]	7.5 (±1.4)[$]	0.48	7.5 (±1.4)[#]	7.6 (±1.9)[#]	0.74						
Hab. drinkers	7.4 (±2.1)[#]	8.3 (±2.8)[#]	0.19	8.3 (±2.8)[$]	8.8 (±2.8)[$]	**0.01**						
Occ. vs. Hab.							0.9 (-0.1-1.9)	0.08	-0.4 (-1.4-0.6)	0.40		
All											0.2 (-0.5-0.9)	0.51

**LDL-apoB,** mg/dL<br>Occ. drinkers<br>Hab. drinkers<br>Occ. vs. Hab.<br>All	63.0 (±11.6)[$]<br>62.0 (±13.4)[#]	63.4 (±13.0)[$]<br>71.1 (±24.4)[#]	0.89<br>0.18	63.4 (±13.0)[#]<br>71.1 (±24.4)[$]	66.4 (±10.8)[#]<br>70.7 (±22.6)[$]	0.44<br>0.88	5.2 (-2.8-13.2)	0.20	3.4 (-4.6-11.3)	0.41	4.3 (-1.4-9.9)	0.14
**ApoB,** mg/dL<br>Occ. drinkers<br>Hab. drinkers<br>Occ. vs. Hab.<br>All	84.9 (±12.9)[$]<br>82.6 (±15.9)[#]	84.4 (±24.4)[$]<br>92.4 (±28.0)[#]	0.85<br>0.21	84.4 (±14.4)[#]<br>92.4 (±28.0)[$]	87.0 (±14.8)[#]<br>93.6 (±26.2)[$]	0.59<br>0.63	6.4 (-3.0-15.8)	0.18	1.40 (-7.9-10.7)	0.77	3.9 (-2.7-10.5)	0.25

Occasional drinkers (n = 10): habitual alcohol intake < 2 drinks (~24 g) per week.
Habitual drinkers (n = 16): habitual alcohol intake ≥ 2 drinks (~24 g) per week, max 1 drink per day in women and 2 drinks per day in men.
[$]Drinking (three weeks).
[#]Abstention (three weeks).
[£]ΔMean1: mean change in occasional drinkers compared with mean change in habitual drinkers in period 1.
[¤]ΔMean2: mean change in occasional drinkers compared with mean change in habitual drinkers in period 2.
*Mean changes in lipids and lipoproteins in drinking participants compared with abstaining participants in both periods, independent of group relationship.
[£,¤,]*Values for drinking periods were subtracted from abstaining, and negative numbers therefore indicate increased levels while drinking compared with abstaining.
Apo: apolipoprotein, -C: cholesterol concentration, Hab.: habitual, LDL: low-density lipoprotein, Occ.: occasional, -PL: phospholipid concentration, -TG: triglyceride concentration.

Table S4. Effects of 1-2 drinks (~12-24 g) per day on NMR measured IDL and VLDL particles and subfractions before and after alcohol intake or abstention and in drinking periods compared with abstention.

	Period 1			Period 2			Period 1		Period 2		Period 1 + 2	
	**Day 0**	**Day 21**	**P**	**Day 22**	**Day 42**	**P**	**ΔMean1**[£]	**P**	**ΔMean2**[¤]	**P**	**ΔMean drinking vs. abstention***	**P**
	Mean (SD)	Mean (SD)		Mean (SD)	Mean (SD)		Mean (95% CI)		Mean (95% CI)		Mean (95% CI)	
**IDL-C,** mg/dL												
Occ. drinkers	7.4 (±2.7)[$]	6.6 (±2.6)[$]	0.31	6.6 (±2.6)[#]	7.1 (±3.4)[#]	0.60						
Hab. drinkers	6.2 (±2.8)[#]	7.8 (±3.8)[#]	0.06	7.8 (±3.8)[$]	8.0 (±4.0)[$]	0.62						
Occ. vs. Hab.							1.9 (0.2-3.7)	**0.03**	0.3 (-1.4-2.0)	0.75		
All											1.1 (-0.1-2.3)	0.08
**IDL-TG,** mg/dL												
Occ. drinkers	9.3 (±2.7)[$]	9.0 (±2.2)[$]	0.60	9.0 (±2.2)[#]	9.2 (±2.8)[#]	0.81						
Hab. drinkers	8.8 (±2.6)[#]	9.2 (±2.4)[#]	0.55	9.2 (±2.4)[$]	9.4 (±2.5)[$]	0.73						
Occ. vs. Hab.							0.6 (-1.0-2.2)	0.45	-0.003 (-1.6-1.6)	0.99		
All											0.3 (-0.8-1.4)	0.60
**VLDL-C,** mg/dL												
Occ. drinkers	10.3 (±4.7)[$]	8.6 (±5.0)[$]	0.30	8.6 (±5.0)[#]	9.1 (±6.8)[#]	0.79						
Hab. drinkers	11.8 (±7.7)[#]	11.6 (±9.8)[#]	0.99	11.6 (±9.8)[$]	14.8 (±9.2)[$]	**0.04**						
Occ. vs. Hab.							1.7 (-2.3-5.8)	0.41	-2.7 (-6.7-1.4)	0.20		
All											-0.5 (-3.3-2.4)	0.75
**VLDL-TG,** mg/dL												
Occ. drinkers	57.5 (±27.2)[$]	50.5 (±23.7)[$]	0.38	50.5 (±23.7)[#]	49.9 (±36.2)[#]	0.95						
Hab. drinkers	66.0 (±42.2)[#]	62.2 (±52.3)[#]	0.71	62.2 (±52.3)[$]	79.8 (±47.4)[$]	0.07						
Occ. vs. Hab.							3.6 (-18.1-25.4)	0.74	-18.2 (-39.8-3.4)	0.10		
All											-7.3 (-22.6-8.1)	0.35
**VLDL-PL,** mg/dL												
Occ. drinkers	18.4 (±8.4)[$]	15.9 (±8.2)[$]	0.33	15.9 (±8.2)[#]	16.1 (±11.4)[#]	0.95						
Hab. drinkers	20.6 (±12.7)[#]	20.1 (±14.8)[#]	0.87	20.1 (±14.8)[$]	24.6 (±14.0)[$]	0.09						
Occ. vs. Hab.							2.1 (-4.4-8.6)	0.53	-4.3 (-10.7-2.2)	0.19		
All											-1.1 (-5.7-3.5)	0.64

Occasional drinkers (n = 10): habitual alcohol intake < 2 drinks (~24 g) per week.
Habitual drinkers (n = 16): habitual alcohol intake ≥ 2 drinks (~24 g) per week, max 1 drink per day in women and 2 drinks per day in men.
[$]Drinking (three weeks).
[#]Abstention (three weeks).
[£]ΔMean1: mean change in occasional drinkers compared with mean change in habitual drinkers in period 1.
[¤]ΔMean2: mean change in occasional drinkers compared with mean change in habitual drinkers in period 2.
*Mean changes in lipids and lipoproteins in drinking participants compared with abstaining participants in both periods, independent of group relationship.
[£,¤,]*Values for drinking periods were subtracted from abstaining, and negative numbers therefore indicate increased levels while drinking compared with abstaining.
-C: cholesterol concentration, Hab.: habitual, IDL: intermediate-density lipoprotein, Occ.: occasional, PL: phospholipid concentration, -TG: triglyceride concentration, -PL: phospholipid concentration, VLDL: very low-density lipoprotein.

Table S5. Effects of 1-2 drinks (~12-24 g) per day on NMR measured apoB-containing particle numbers before and after alcohol intake or abstention and in drinking periods compared with abstention.

	Period 1			Period 2			Period 1		Period 2		Period 1 + 2	
	**Day 0**	**Day 21**	**P**	**Day 22**	**Day 42**	**P**	**ΔMean1**[£]	**P**	**ΔMean2**[¤]	**P**	**ΔMean drinking vs. abstention***	**P**
	Mean (SD)	Mean (SD)		Mean (SD)	Mean (SD)		Mean (95% CI)		Mean (95% CI)		Mean (95% CI)	
**LDL-P, nmol**/L												
Occ. drinkers	1146 (±210)[$]	1153 (±237)[$]	0.89	1153 (±237)[#]	1208 (±196)[#]	0.44						
Hab. drinkers	1127 (±244)[#]	1292 (±443)[#]	0.18	1292 (±443)[$]	1286 (±412)[$]	0.88						
Occ. vs. Hab.							95 (-51-241)	0.20	61 (-84-206)	0.41		
All											78 (-25-181)	0.14
**LDL_1-P, nmol**/L												
Occ. drinkers	268 (±67)[$]	274 (±76)[$]	0.77	274 (±76)[#]	293 (±48)[#]	0.40						
Hab. drinkers	267 (±78)[#]	307 (±149)[#]	0.41	307 (±149)[$]	308 (±129)[$]	0.93						
Occ. vs. Hab.							13 (-32-57)	0.57	18 (-26-62)	0.43		
All											15 (-16-47)	0.34
**LDL_2-P, nmol**/L												
Occ. drinkers	168 (±33)[$]	175 (±37)[$]	0.56	175 (±37)[#]	178 (±27)[#]	0.82						
Hab. drinkers	161 (±41)[#]	196 (±83)[#]	0.23	196 (±83)[$]	179 (±82)[$]	0.39						
Occ. vs. Hab.							9 (-19-37)	0.53	12 (-15-40)	0.39		
All											11 (-9-30)	0.29
**LDL_3-P, nmol**/L												
Occ. drinkers	160 (±33)[$]	161 (±34)[$]	0.94	161 (±34)[#]	163 (±34)[#]	0.87						
Hab. drinkers	154 (±43)[#]	178 (±75)[#]	0.36	178 (±75)[$]	186 (±69)[$]	0.26						
Occ. vs. Hab.							12 (-14-39)	0.36	-7 (-33-20)	0.62		
All											3 (-16-22)	0.76
**LDL_4-P, nmol**/L												
Occ. drinkers	157 (±34)[$]	157 (±33)[$]	0.97	157 (±33)[#]	157 (±38)[#]	0.98						
Hab. drinkers	152 (±44)[#]	173 (±67)[#]	0.34	173 (±67)[$]	179 (±66)[$]	0.38						
Occ. vs. Hab.							13 (-12-38)	0.33	-6 (-31-19)	0.62		
All											3 (-14-21)	0.73
**LDL_5-P, nmol**/L												
Occ. drinkers	135 (±35)[$]	131 (±31)[$]	0.43	131 (±31)[#]	130 (±37)[#]	0.94						
Hab. drinkers	129 (±42)[#]	145 (±55)[#]	0.26	145 (±55)[$]	152 (±57)[$]	0.28						
Occ. vs. Hab.							16 (-4-36)	0.12	-8 (-28-12)	0.42		
All											4 (-10-18)	0.60
**LDL_6-P, nmol**/L												
Occ. drinkers	187 (±52)[$]	165 (±37)[$]	0.15	165 (±37)[#]	156 (±43)[#]	0.55						
Hab. drinkers	161 (±76)[#]	161 (±102)[#]	0.89	161 (±102)[$]	194 (±150)[$]	0.06						
Occ. vs. Hab.							23 (-20-65)	0.29	-42 (-84-0.3)	0.05		
All											-9 (-39-20)	0.53
**VLDL-P, nmol**/L												
Occ. drinkers	103 (±38)[$]	90 (±45)[$]	0.41	90 (±45)[#]	89 (±50)[#]	0.93						
Hab. drinkers	109 (±47)[#]	102 (±52)[#]	0.46	102 (±52)[$]	115 (±49)[$]	0.19						
Occ. vs. Hab.							5 (-25-34)	0.77	-14 (-44-15)	0.35		
All											-5 (-26-16)	0.65
**IDL-P, nmol**/L												
Occ. drinkers	106 (±30)[$]	101 (±23)[$]	0.41	101 (±23)[#]	101 (±33)[#]	0.94						
Hab. drinkers	94 (±26)[#]	104 (±30)[#]	0.12	104 (±30)[$]	102 (±29)[$]	0.66						
Occ. vs. Hab.							13 (-2-28)	0.10	3 (-13-18)	0.74		
All											8 (-3-18)	0.16

Occasional drinkers (n = 10): habitual alcohol intake < 2 drinks (~24 g) per week.
Habitual drinkers (n = 16): habitual alcohol intake ≥ 2 drinks (~24 g) per week, max 1 drink per day in women and 2 drinks per day in men.
[$]Drinking (three weeks).
[#]Abstention (three weeks).
[£]ΔMean1: mean change in occasional drinkers compared with mean change in habitual drinkers in period 1.
[¤]ΔMean2: mean change in occasional drinkers compared with mean change in habitual drinkers in period 2.
*Mean changes in lipids and lipoproteins in drinking participants compared with abstaining participants in both periods, independent of group relationship.
[£,¤,]*Values for drinking periods were subtracted from abstaining, and negative numbers therefore indicate increased levels while drinking compared with abstaining.

Hab.: habitual, IDL: intermediate-density lipoprotein, LDL: low-density lipoprotein, Occ.: occasional, -P: particle number concentration, VLDL: very low-density lipoprotein.

Table S6. Effects of 1 drink per day (~12 g) on NMR measured HDLs, apoA1, and HDL subfractions before and after alcohol intake or abstention and in drinking periods compared with abstention in women.

	**Period 1**			**Period 2**			**Period 1**		**Period 2**		**Period 1 + 2**	
	**Day 0**	**Day 21**	**P**	**Day 22**	**Day 42**	**P**	**ΔMean1**[£]	**P**	**ΔMean2**[¤]	**P**	**ΔMean drinking vs. abstention***	**P**
	Mean (SD)	Mean (SD)		Mean (SD)	Mean (SD)		Mean (95% CI)		Mean (95% CI)		Mean (95% CI)	
**HDL-C,** mg/dL<br>Occ. drinkers<br>Hab. drinkers<br>Occ. vs. Hab.<br>All	61.8 (±15.2)[$]<br>63.4 (±19.9)[#]	63.0 (±9.8)[$]<br>62.4 (±20.2)[#]	0.76<br>0.76	63.0 (±9.8)[#]<br>62.4 (±20.2)[$]	63.5 (±18.5)[#]<br>65.3 (±19.0)[$]	0.94<br>0.20	-2.4 (-11.9-7.1)	0.62	-2.5 (-11.8-6.8)	0.60	-2.4 (-9.1-4.2)	0.47
**HDL$_{2b}$-C,** mg/dL<br>Occ. drinkers<br>Hab. drinkers<br>Occ. vs. Hab.<br>All	24.5 (±13.0)[$]<br>26.6 (±15.6)[#]	24.7 (±7.3)[$]<br>27.7 (±16.5)[#]	0.95<br>0.47	24.7 (±7.3)[#]<br>27.7 (±16.5)[$]	27.7 (±15.3)[#]<br>28.2 (±15.1)[$]	0.41<br>0.72	0.9 (-5.4-7.1)	0.79	2.4 (-3.8-8.6)	0.44	1.6 (-2.8-6.0)	0.47
**HDL$_{2a}$-C,** mg/dL<br>Occ. drinkers<br>Hab. drinkers<br>Occ. vs. Hab.<br>All	25.0 (±5.0)[$]<br>24.9 (±4.8)[#]	25.7 (±4.2)[$]<br>23.9 (±4.0)[#]	0.40<br>0.13	25.7 (±4.2)[#]<br>23.9 (±4.0)[$]	24.9 (±4.9)[#]<br>24.9 (±3.7)[$]	0.60<br>0.21	-1.9 (-4.5-0.7)	0.15	-1.9 (-4.5-0.7)	0.15	-1.9 (-3.7-[-0.06])	**0.04**
**HDL$_{3}$-C,** mg/dL<br>Occ. drinkers<br>Hab. drinkers<br>Occ. vs. Hab.<br>All	18.0 (±2.7)[$]<br>17.4 (±1.0)[#]	19.1 (±1.9)[$]<br>17.3 (±2.0)[#]	0.22<br>0.63	19.1 (±1.9)[#]<br>17.3 (±2.0)[$]	17.5 (±2.2)[#]<br>16.8 (±2.7)[$]	**0.03**<br>0.58	-1.4 (-3.3-0.5)	0.14	-1.1 (-3.0-0.7)	0.23	-1.3 (-2.6-0.04)	0.057
**HDL$_{2a}$-PL,** mg/dL<br>Occ. drinkers<br>Hab. drinkers<br>Occ. vs. Hab.<br>All	50.7 (±14.7)[$]<br>48.4 (±10.4)[#]	51.4 (±11.0)[$]<br>46.8 (±8.4)[#]	0.72<br>0.30	51.4 (±11.0)[#]<br>46.8 (±8.4)[$]	49.6 (±12.5)[#]<br>47.4 (±5.0)[$]	0.59<br>0.78	-2.0 (-8.2-4.2)	0.53	-2.4 (-8.4-3.7)	0.44	-2.2 (-6.5-2.1)	0.32
**ApoA1,** mg/dL<br>Occ. drinkers<br>Hab. drinkers<br>Occ. vs. Hab.<br>All	157.9 (±30.7)[$]<br>155.8 (±29.1)[#]	159.8 (±24.8)[$]<br>150.1 (±28.8)[#]	0.71<br>0.23	159.8 (±24.8)[#]<br>150.1 (±28.8)[$]	152.8 (±33.4)[#]<br>156.8 (±24.2)[$]	0.49<br>0.15	-8.2 (-24.8-8.3)	0.33	-13.7 (-30.0-2.5])	0.10	-11.0 (-22.6-0.6)	0.063
**HDL-apoA1,** mg/dL<br>Occ. drinkers<br>Hab. drinkers<br>Occ. vs. Hab.<br>All	136.5 (±27.6)[$]<br>136.2 (±29.0)[#]	138.5 (±21.8)[$]<br>131.1 (±26.7)[#]	0.68<br>0.25	138.5 (±21.8)[#]<br>131.1 (±26.7)[$]	134.9 (±29.5)[#]<br>138.6 (±25.1)[$]	0.69<br>0.08	-6.9 (-21.5-7.7)	0.35	-11.1 (-25.4-3.2)	0.13	-9.0 (-19.2-1.2)	0.08
**HDL$_{2b}$-apoA1,**<br>mg/dL<br>Occ. drinkers<br>Hab. drinkers<br>Occ. vs. Hab.<br>All	36.1 (±17.6)[$]<br>40.0 (±21.5)[#]	35.8 (±10.7)[$]<br>40.4 (±23.3)[#]	0.95<br>0.50	35.8 (±10.7)[#]<br>40.4 (±23.3)[$]	39.4 (±20.9)[#]<br>42.8 (±19.6)[$]	0.49<br>0.40	1.6 (-7.5-10.7)	0.73	1.2 (-7.7-10.2)	0.79	1.4 (-4.9-7.8)	0.66
**HDL$_{2a}$-apoA1,**<br>mg/dL<br>Occ. drinkers<br>Hab. drinkers<br>Occ. vs. Hab.<br>All	52.8 (±13.8)[$]<br>51.2 (±9.9)[#]	53.6 (±11.4)[$]<br>48.1 (±8.1)[#]	0.59<br>0.051	53.6 (±11.4)[#]<br>48.1 (±8.1)[$]	50.8 (±12.2)[#]<br>50.0 (±5.2)[$]	0.36<br>0.29	-3.5 (-9.0-1.0)	0.20	-4.7 (-10.0-0.6)	0.08	-4.1 (-7.9-[-0.3])	**0.03**
**HDL$_{3}$-apoA1,** mg/dL<br>Occ. drinkers<br>Hab. drinkers<br>Occ. vs. Hab.<br>All	60.0 (±10.2)[$]<br>55.2 (±5.1)[#]	61.3 (±8.5)[$]<br>54.4 (±6.3)[#]	0.49<br>0.39	61.3 (±8.5)[#]<br>54.4 (±6.3)[$]	56.3 (±9.8)[#]<br>55.2 (±6.4)[$]	0.051<br>0.69	-3.3 (-8.6-2.0)	0.22	-5.9 (-11.0-[-0.7])	**0.027**	-4.6 (-8.3-[-0.9])	**0.016**
**ApoB/apoA1,** mg/dL<br>Occ. drinkers<br>Hab. drinkers<br>Occ. vs. Hab.<br>All	0.56 (±0.13)[$]<br>0.47 (±0.07)[#]	0.54 (±0.14)[$]<br>0.59 (±0.20)[#]	0.66<br>**0.02**	0.54 (±0.14)[#]<br>0.59 (±0.20)[$]	0.58 (±0.13)[#]<br>0.55 (±0.15)[$]	0.33<br>**0.04**	0.09 (0.01-0.16)	**0.02**	0.09 (0.02-0.16)	**0.01**	0.09 (0.04-0.14)	**0.001**
**LDL-apoB,** mg/dL<br>Occ. drinkers<br>Hab. drinkers<br>Occ. vs. Hab.<br>All	63.0 (±11.6)[$]<br>52.8 (±8.5)[#]	63.4 (±13.0)[$]<br>66.1 (±24.8)[#]	0.89<br>0.14	63.4 (±13.0)[#]<br>66.1 (±24.8)[$]	66.4 (±10.8)[#]<br>63.7 (±24.5)[$]	0.44<br>0.35	5.5 (-2.1-13.2)	0.15	5.4 (-2.0-12.9)	0.15	5.5 (0.2-10.8)	**0.04**
**LDL-P, nmol**/L<br>Occ. drinkers<br>Hab. drinkers<br>Occ. vs. Hab.<br>All	1146 (±210)[$]<br>961 (±154)[#]	1153 (±237)[$]<br>1203 (±451)[#]	0.89<br>0.14	1153 (±237)[#]<br>1203 (±451)[$]	1208 (±196)[#]<br>1159 (±446)[$]	0.44<br>0.35	101 (-38-239)	0.15	99 (-37-234)	0.15	100 (3-197)	**0.04**

**ApoB,** mg/dL<br>Occ. drinkers<br>Hab. drinkers<br>Occ. vs. Hab.<br>All	84.9 (±12.9)[$]<br>72.0 (±9.3)[#]	84.4 (±14.4)[$]<br>87.0 (±28.4)[#]	0.85<br>0.15	84.4 (±14.4)[#]<br>87.0 (±28.4)[$]	87.0 (±14.8)[#]<br>85.4 (±28.0)[$]	0.59<br>0.54	7.2 (-2.0-16.4)	0.12	4.3 (-4.7-13.3)	0.35	5.7 (-0.7-12.2)	0.08
**LDL-TG,** mg/dL<br>Occ. drinkers<br>Hab. drinkers<br>Occ. vs. Hab.<br>All	16.1 (±3.3)[$]<br>13.7 (±3.7)[#]	15.9 (±2.1)[$]<br>16.0 (±4.3)[#]	0.83<br>0.13	15.9 (±2.1)[#]<br>16.0 (±4.3)[$]	16.8 (±3.6)[#]<br>16.6 (±3.4)[$]	0.38<br>0.54	2.1 (-0.4-4.6)	0.10	0.2 (-2.2-2.7)	0.85	1.2 (-0.6-2.9)	0.19
**LDL$_1$-TG,** mg/dL<br>Occ. drinkers<br>Hab. drinkers<br>Occ. vs. Hab.<br>All	5.4 (±1.9)[$]<br>4.3 (±1.7)[#]	5.2 (±0.9)[$]<br>5.3 (±1.9)[#]	0.76<br>0.20	5.2 (±0.9)[#]<br>5.3 (±1.9)[$]	5.5 (±1.7)[#]<br>5.6 (±1.7)[$]	0.50<br>0.52	1.0 (-0.3-2.3)	0.12	0.1 (-1.2-1.3)	0.94	0.5 (-0.4-1.4)	0.24

Occasional drinkers (n = 10): habitual alcohol intake < 2 drinks (~24 g) per week.
Habitual drinkers (n = 16): habitual alcohol intake ≥ 2 drinks (~24 g) per week, max 1 drink per day in women and 2 drinks per day in men.
[$]Drinking (three weeks).
[#]Abstention (three weeks).
[£]ΔMean1: mean change in occasional drinkers compared with mean change in habitual drinkers in period 1.
[¤]ΔMean2: mean change in occasional drinkers compared with mean change in habitual drinkers in period 2.
*Mean changes in lipids and lipoproteins in drinking participants compared with abstaining participants in both periods, independent of group relationship.
[£,¤,]*Values for drinking periods were subtracted from abstaining, and negative numbers therefore indicate increased levels while drinking compared with abstaining.
Apo: apolipoprotein, -C: cholesterol concentration, Hab.: habitual, HDL: high-density lipoprotein, LDL: low-density lipoprotein, Occ.: occasional, -P: particle number concentration, -PL: phospholipid concentration, -TG: triglyceride concentration.
